# Genetic targeting and anatomical registration of neuronal populations in the zebrafish brain with a new set of BAC transgenic tools

**DOI:** 10.1038/s41598-017-04657-x

**Published:** 2017-07-12

**Authors:** Dominique Förster, Irene Arnold-Ammer, Eva Laurell, Alison J. Barker, António M. Fernandes, Karin Finger-Baier, Alessandro Filosa, Thomas O. Helmbrecht, Yvonne Kölsch, Enrico Kühn, Estuardo Robles, Krasimir Slanchev, Tod R. Thiele, Herwig Baier, Fumi Kubo

**Affiliations:** 1 Max Planck Institute of Neurobiology, Department Genes - Circuits - Behavior, Am Klopferspitz 18, D-82152 Martinsried, Germany; 2 Max-Delbrück Center for Molecular Medicine, Berlin, Germany; 3 Department of Biological Sciences, Purdue University, West Lafayette, USA; 4 Department of Biological Sciences, University of Toronto Scarborough, Toronto, Canada

## Abstract

Genetic access to small, reproducible sets of neurons is key to an understanding of the functional wiring of the brain. Here we report the generation of a new Gal4- and Cre-driver resource for zebrafish neurobiology. Candidate genes, including cell type-specific transcription factors, neurotransmitter-synthesizing enzymes and neuropeptides, were selected according to their expression patterns in small and unique subsets of neurons from diverse brain regions. BAC recombineering, followed by Tol2 transgenesis, was used to generate driver lines that label neuronal populations in patterns that, to a large but variable extent, recapitulate the endogenous gene expression. We used image registration to characterize, compare, and digitally superimpose the labeling patterns from our newly generated transgenic lines. This analysis revealed highly restricted and mutually exclusive tissue distributions, with striking resolution of layered brain regions such as the tectum or the rhombencephalon. We further show that a combination of Gal4 and Cre transgenes allows intersectional expression of a fluorescent reporter in regions where the expression of the two drivers overlaps. Taken together, our study offers new tools for functional studies of specific neural circuits in zebrafish.

## Introduction

Deciphering the circuitry of the brain requires experimental strategies that allow monitoring and manipulating the function of individual neurons within larger, genetically defined populations. To this end, numerous genetically encoded sensors and actuators are available to neurobiologists^1, 2^, but their utility strongly depends on the precision by which these effectors can be genetically targeted to small subsets of cells. Binary expression systems, like the Gal4/UAS and the Cre/lox system, are to date the most popular tools to target gene expression to defined cell populations, while retaining flexibility of driver and effector combinations^3, 4^. Further, engineering a combined Gal4-plus-Cre system in which a UAS-linked reporter is flanked by loxP sites followed by a second transgene on the same construct allows to restrict the expression of that second transgene to cells in which Gal4 and Cre expression patterns overlap. While this intersectional strategy was first implemented in zebrafish by Satou *et al*.^5^, a related approach was pioneered for single-cell labeling by Sato *et al*.^6^.

Several thousand transgenic zebrafish lines have been generated using short enhancer/promoter sequences from known genes or by unbiased enhancer trap screens^7–12^. As single enhancer sites typically do not regulate the complex spatial and temporal aspects of expression, these lines often do not faithfully recapitulate the expression pattern of the endogenous gene^13, 14^. Recent advances in transgenesis techniques have made it possible to more reliably reproduce the endogenous expression of the gene of interest by using the larger gene regulatory regions (50–300 kb) contained in bacterial artificial chromosomes (BACs). State-of-the-art protocols for efficient application of BAC recombineering and transgenesis in zebrafish have been established^15, 16^, paving the way for larger-scale approaches to generate reliable tools for targeted gene expression. Here we report the generation, characterization and comparison of 58 BAC-derived Gal4 and Cre constructs. This screen has led to the production of 22 new transgenic zebrafish lines, each of which allows genetic access to unique neuronal subpopulations.

## Results and Discussion

### Transgenesis by BAC recombineering yields 22 stable zebrafish lines

**Figure 1:**
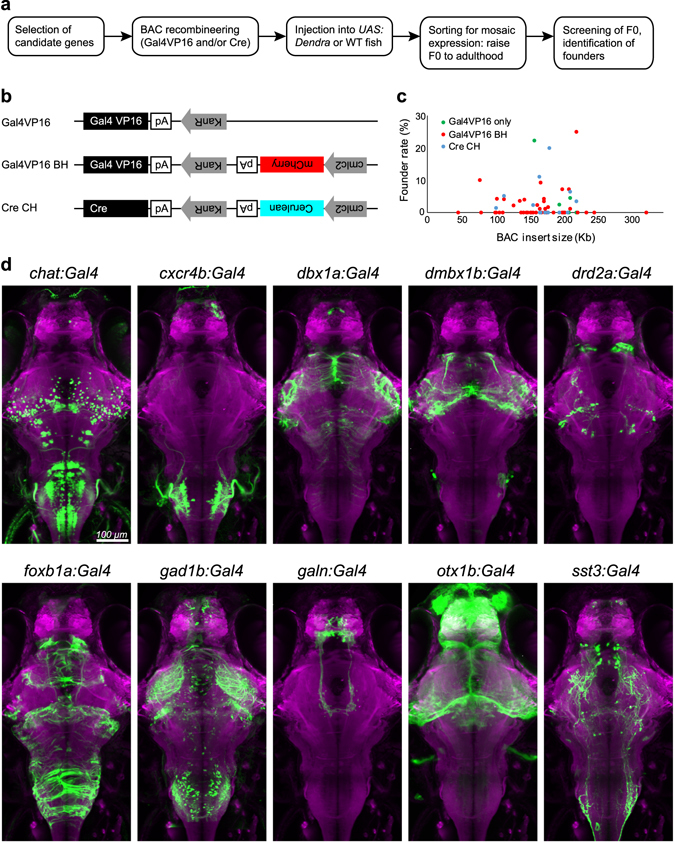
A candidate-based BAC screen identifies new genetic tools for targeting neuronal subpopulations. (**a**) Workflow to establish BAC transgenic fish lines. WT, wild-type. (**b**) Plasmid constructs used for BAC recombineering. *KanR*, kanamycine-resistant gene; *pA*, polyadenylation signal. (**c**) Relationship between the genomic insert length of all BAC constructs (7 *Gal4VP16* BACs, 39 *Gal4VP16-Bleeding Heart* BACs, and 12 *Cre-Cold Heart* BACs) and their founder rate (percentage of germline founders out of total adult fish screened). Note that the founder rate is not correlated with the genomic insert length (R-squared = 0.0007). (**d**) Dorsal view of 6 dpf old larval brains showing the live expression pattern of ten selected transgenic Gal4 lines (green; Dendra-kras, GCaMP6s or EGFP). Brains have been registered via co-expression of *HuC:lynTagRFP-T* (magenta). Scale bar, 100 µm.

To generate an array of transgenic lines that label diverse subsets of neuronal populations, we selected candidate genes primarily based on their published spatiotemporal expression patterns (Fig. 1a and Table 1). A large fraction of these genes had previously been shown by RNA *in situ* hybridization to be transcribed in small populations of retinal ganglion cells (RGCs) and/or tectal neurons. In addition, we were interested in neurons that share the transmitter GABA or acetylcholine across the brain and therefore included genes that encode for transmitter-synthesizing enzymes. A third category was genes encoding neuropeptides, since these are some of the most specific markers of defined cell types in the central nervous system^17, 18^.

**Table 1:** List of Gal4VP16 and Gal4VP16-Bleeding Heart constructs and transgenic lines.

Gal4VP16 only							
Gene name	Expression pattern	BAC clone #	BAC insert size (Kb)	# Fish screened	# Positive founders	Founder rate (%)	Allele number
arl4ca	RGCs	DKEY-16H18	208	22	0	0	
cacnb3b	RGCs	DKEY-180E8	210	32	0	0	
**chata**	**OT, HB, spinal cord**	**DKEY-194K24**	**212**	**22**	**1**	**4.5**	**mpn202, 204×**
chodl	FB, tegmentum, OT, HB	CH211-209C15	200	22	0	0	
**cxcr4b**	**RGCs, FB, HB**	**DKEY-260L22**	**196**	**42**	**1**	**2.4**	**mpn206**
**dmbx1b**	**OT, HB**	**CH211-39M11**	**159**	**9**	**2**	**22.2**	**mpn207**
grin2ab	RGCs	DKEY-90H7	221	34	0	0	
**Gal4VP16 Bleeding Heart (clmc2:mCherry)**							
**Gene name**	**Expression pattern**	**BAC clone #**	**BAC insert size (Kb)**	**# Fish screened**	**# Positive founders**	**Founder rate (%)**	**Allele number**
adcyap1a	habenula, HB	CH73-310J3	107	28	0	0	
arl4ca	RGCs	DKEY-16H18	208	12	0	0	
bcl6a	cerebellum, OT, retina	DKEY-253J8	174	8	0	0	
cabp7b	OT, tegmentum, cerebellum	DKEY-87A16	161	108	0	0	
cacnb3b*	**RGCs**	**DKEY-180E8**	**210**	**14**	**1**	**7.1**	
cadm4	retina, FB, MB, HB	DKEY-178J22	204	71	0	0	
cart2*	**FB, HB, tegmentum**	**DKEY-114P5**	**175**	**91**	**1**	**1.1**	
chata*	**OT, HB, spinal cord**	**DKEY-194K24**	**212**	**85**	**1**	**1.2**	
chodl	FB, tegmentum, OT, HB	CH211-209C15	200	56	0	0	
coch	unknown	CH211-29J20	154	13	0	0	
**dbx1a**	**FB, MB, HB**	**CH211-271F10**	**128**	**45**	**1**	**2.2**	**mpn210**
drd1b	FB, hypothalamus, HB	CH211-148O10	147	63	0	0	
drd2a	FB, HB, OT, spinal cord	DKEY-50H16	325	41	0	0	
**drd2a**	**FB, HB, OT, spinal cord**	**CH73-335E14**	**103**	**24**	**1**	**4.2**	**mpn211**
**foxb1a**	**FB, HB, OT**	**CH211-2C17**	**144**	**50**	**2**	**4.0**	**mpn212**
foxb1b	OT, HB	CH211-92B21	177	59	0	0	
**gad1b**	**FB, HB, OT**	**CH211-24M22**	**168**	**54**	**5**	**9.3**	**mpn155, 200, 201**
gad2*	**FB, hypothalamus, pretectum**	**CH211-37E10**	**167**	**88**	**1**	**1.1**	
**galn**	**hypothalamus, preoptic area**	**CH211-103A19**	**78**	**10**	**1**	**10.0**	**mpn213**
grin2aa	retina	DKEY-255O2	202	29	0	0	
grin2ab*	**RGCs**	**DKEY-90H7**	**221**	**4**	**1**	**25.0**	
**id2b**	**retina, OT, HB**	**CH211-175H7**	**137**	**28**	**1**	**3.6**	**mpn215**
**lhx2b**	**FB, MB, HB, retina**	**CH211-159C13**	**173**	**57**	**2**	**3.5**	**mpn216**
**lhx9**	**FB, MB, HB**	**DKEY-121A9**	**200**	**56**	**4**	**7.1**	**mpn203, 205**
lrrtm4l2	MHB, HB, retina	CH73-359G19	102	45	0	0	
nos1	MB, spinal cord	CH73-385P12	112	34	0	0	
opn4b	retina, FB	DKEY-156P15	223	58	0	0	
**otx1b**	**FB, MB, OT**	**DKEY-209N21**	**173**	**48**	**2**	**4.2**	**mpn217**
pcp4a	FB, HB	CH211-231M12	149	57	0	0	
penka	FB, MB, HB, spinal cord	CH211-189N20	149	28	0	0	
rorb	retina, MB	DKEY-196E24	80	12	0	0	
sdk2b	unknown	DKEY-172F4	169	10	0	0	
slc6a4a	pretectum, raphe	DKEY-263M15	157	8	0	0	
**slit1a**	**retina, OT, HB**	**DKEY-118N13**	**114**	**74**	**3**	**4.1**	**mpn218**
sp5l	OT	DKEY-156F14	187	19	0	0	
**sst3**	**FB, MB**	**DKEY-265F18**	**163**	**44**	**1**	**2.3**	**mpn219**
tac1	FB, HB	CH211-51C11	143	28	0	0	
**tbx20**	**RGCs, tegmentum, HB**	**CH211-132C11**	**179**	**117**	**2**	**1.7**	**mpn220**
tmem200a	FB, OT, MB, RGCs	DKEY-252D12	139	20	0	0	

Seven Gal4VP16 and 39 Gal4VP16-Bleeding Heart BAC constructs that were generated and validated to express Gal4VP16 after transient injection. Founder rate is defined as a percentage of germline founders out of total adult fish screened. Bold texts indicate the constructs from which Gal4VP16-expressing transgenic line(s) were isolated. FB, forebrain; MB, midbrain; HB, hindbrain; RGCs, retinal ganglion cells; OT, optic tectum; *lines discontinued; ^×^second line identified in an additional round of screening.

For each gene, we chose a BAC clone that contains both upstream and downstream sequences of the translation start site to include transcription regulatory elements on both sides of the coding sequence. BAC recombineering was performed according to the protocol of Bussmann and Schulte-Merker, which is a plasmid-based technique employing an arabinose-inducible homologous recombination^15^. The method was slightly modified by adding a stable fluorescent marker to the construct, which labels cardiac muscle cells for ease of screening and subsequent re-identification. The red-fluorescent marker “bleeding heart” (BH, *cmlc2:mCherry*) was linked to Gal4VP16 constructs, and “cold heart” (CH, *cmlc2:Cerulean*) was linked to Cre (Fig. 1b). In addition, a small subset of Gal4VP16 lines was generated without heart marker.

Recombineered BAC constructs were injected into fertilized eggs obtained from *UAS:Dendra-kras* transgenic fish or from wild-type fish. Potential F0 founders were selected at larval stages for their expression of the transgene and raised to fertility. Specifically, we selected F0 larvae that expressed Dendra (for Gal4VP16 and Gal4VP16-BH), the “bleeding heart” marker (for Gal4VP16-BH) or the “cold heart” marker (for Cre-CH). When the F0 fish had reached adulthood, they were crossed to wild type, *UAS:Dendra* or *UAS:intersec* (see below) and their F1 progeny were screened for inherited transgene expression in the expected pattern. Germline transformation and mosaicism rates were similar to other Tol2 based BAC transgenesis approaches previously reported^5, 15^.

Out of 58 BAC constructs that were confirmed to drive expression of Gal4VP16, Gal4VP16-BH, or Cre-CH, we isolated 22 stable transgenic lines. The results are summarized in Tables 1 and 2. The success rate for obtaining stable transgenic lines was 42.9% (3/7 genes), 43.6% (17/39 genes) and 50% (6/12 genes) for Gal4VP16, Gal4VP16-BH and Cre-CH, respectively. For those stable transgenic lines, the percentage of germline transgenic founders out of all adult fish screened (“founder rate”) ranged from 1.1% to 25% (average 6.3%). In general, the founder rates of different driver constructs derived from the same BAC (Gal4VP16 only, Gal4VP16-BH and Cre-CH) were found to be similar, with one exception being *grin2ab* (25% and 3.4% for the Gal4-BH and Cre-CH construct, respectively). Consistent with a previous report^15^, we did not find a clear correlation between transgenesis efficiency and genomic insert length of the BACs (Fig. 1c).

**Table 2:** List of *Cre-Cold Heart* constructs and transgenic lines.

Cre Cold Heart (clmc2:Cerulean)							
Gene name	Expression pattern	BAC clone #	BAC insert size (Kb)	# Fish screened	# Positive founders	Founder rate (%)	Allele number
arl4ca	RGCs	DKEY-16H18	208	75	0	0	
**atoh7**	**RGCs**	**DKEY-111E19**	**181**	**10**	**2**	**20.0**	**mpn221**
cart2	FB, HB, tegmentum	DKEY-114P5	175	12	0	0	
chata*	**OT, HB, spinal cord**	**DKEY-194K24**	**212**	**62**	**4**	**6.5**	
cxcr4b	RGCs, FB, HB	DKEY-260L22	196	80	0	0	
gad1b	FB, HB, OT	CH211-24M22	168	2	0	0	
gad2	FB, hypothalamus, pretectum	CH211-37E10	167	34	0	0	
grin2ab*	**RGCs**	**DKEY-90H7**	**221**	**58**	**2**	**3.4**	
isl2b*	**RGCs, cranial ganglia**	**DKEY-73M9**	**157**	**88**	**2**	**2.3**	
otx1b	FB, MB, OT	DKEY-209N21	173	N/A	N/A	N/A	
**slit1a**	**retina, OT, HB**	**DKEY-118N13**	**114**	**58**	**3**	**5.2**	**mpn222**
tbx20	RGCs, tegmentum, HB	CH211-132C11	179	4	0	0	
**th**	**FB, hypothalamus, HB**	**CH211-77O7**	**166**	**36**	**4**	**11.1**	**mpn223**

12 *Cre-Cold Heart* BAC constructs have been generated and validated to express Cre after transient injection. E﻿xpression p﻿attern is derived from previously reported expression data. Founder rate is defined as a percentage of germline founders out of total adult fish screened. Bold texts indicate the constructs from which CH-positive transgenic line(s) were isolated. FB, forebrain; MB, midbrain; HB, hindbrain; RGCs, retinal ganglion cells; OT, optic tectum; *lines discontinued. N/A, line has not been screened for founders. *otx1b:Cre* BAC was generated without the Cold Heart cassette.

### Gal4VP16 and Cre drivers give access to defined neuronal subsets by largely recapitulating endogenous gene expression

**Figure 2:**
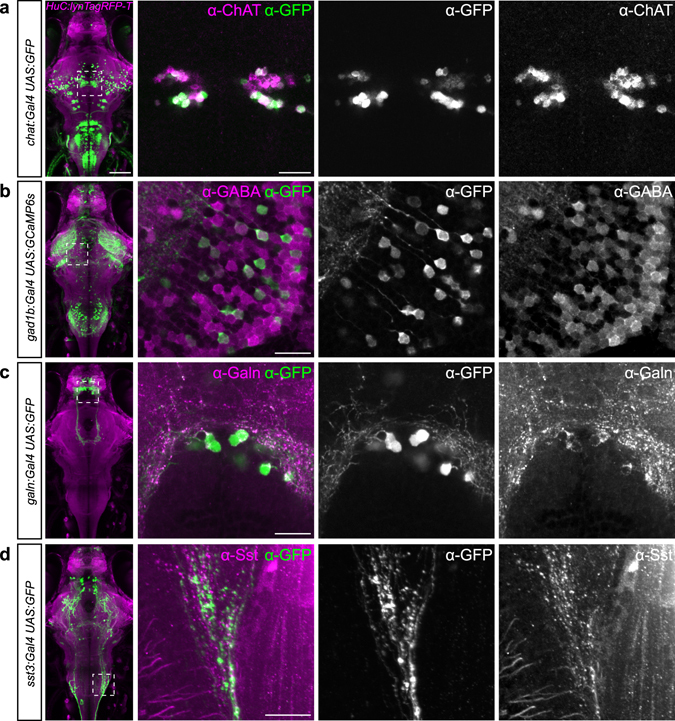
New transgenic lines largely recapitulate endogenous gene expression. (**a**–**d**) Antibody stainings of 6 dpf old larvae against GFP (green) and ChAT (**a**), GABA (**b**), Galanin (**c**) or Somatostatin (**d**), respectively (magenta). Genotypes are indicated on the left. Whole-brain images on the left show live expression pattern (lynTagRFP-T in magenta), outlining the location of the magnified regions on the right. Scale bar, 100 µm for overview, 20 µm for magnified images.

In accord with the RNA expression patterns of the chosen genes, the established Gal4 transgenic lines label different subsets of neurons in the brain (Fig. 1d and Supplementary Video 1–11). To determine to what extent the BAC-driven Gal4 patterns recapitulate endogenous gene expression, we compared expression of a UAS-linked reporter (*UAS:GFP* or *UAS:GCaMP6s*) to the distribution of the selected genes by antibody staining. The *Tg*(*chat:Gal4*) and *Tg*(*gad1b:Gal4*) lines labeled cells in several brain areas that were positive for choline acetyltransferase (ChAT) and GABA, respectively (Fig. 2a,b). Out of all ChAT- or GABA-positive cells, a little less than half visibly expressed the UAS-linked reporter in each line (48 ± 24%, n = 105 of 231 cells out of two larvae in *Tg*(*chat:Gal4*), and 42 ± 15%, n = 150 of 355 cells out of four larvae in *Tg*(*gad1b:Gal4*)). In *Tg*(*galn:Gal4*) and *Tg*(*sst3:Gal4*) lines, antibody staining greatly overlapped with, or was identical to, the Gal4-driven reporter expression (94 ± 4%, n = 88 of 94 cells out of two larvae in *Tg*(*galn:Gal4*) line) (Fig. 2c,d). Differences in protein localizations in *Tg*(*galn:Gal4*) *and Tg*(*sst3:Gal4*) lines can be explained by the transport into axon terminals of the Galn and Sst proteins, both of which encode neuropeptide transmitters^19, 20^ versus the cytosolic localization of GFP. It is also possible that some of the signal from the Galn and Sst antibodies is derived from extracellular localization of these secreted factors. These results with four transgenes, for which cross-reactive antibodies are available, suggest that the BAC transgenic Gal4 lines largely reproduce the endogenous gene expression pattern and thus provide genetic access to the labeled neuronal populations. Individual transgenic lines should be tested for recapitulation of endogenous gene expression patterns for each study at hand.

**Figure 3:**
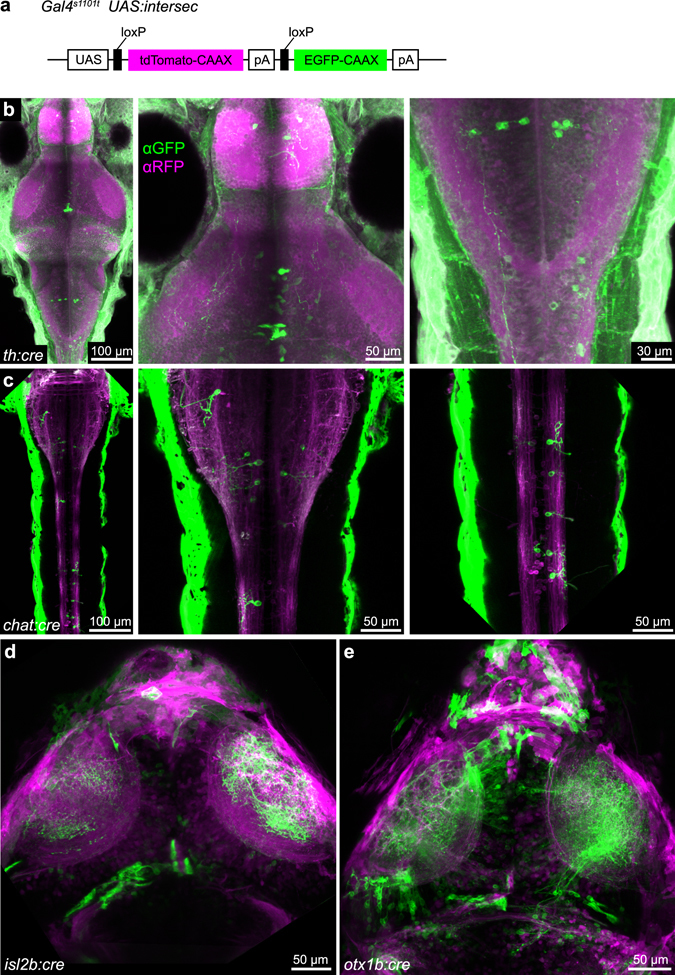
Cre-expressing BAC constructs allow intersectional genetics. (**a**) *UAS:intersec* construct for intersectional genetic approach. A pan-neuronal Gal4 line (*Gal4s1101t*) was used in this figure. (**b**) Antibody staining against GFP and RFP reveals highly variegated, transgenic expression of *th:cre* stable transgenic line. (**c**–**e**) Live imaging showing transient expression of *chat:cre* (**c**), *isl2b:cre* (**d**) and *otx1b:cre* (**e**).

We also confirmed expression of our Cre constructs, either transiently or in stable transgenic lines, using a Cre reporter line named *UAS:intersec* (Fig. 3a). The *UAS:intersec* transgene is designed to drive the expression of the reporter gene in cells in which Gal4 and Cre overlap. This enables intersectional genetic approaches as reported previously^5, 21^. Taken together, the BAC transgenic driver lines established here allow experimental access to small neuronal populations, either in binary (Gal4/UAS) or ternary (Cre/Gal4/*UAS:intersec*) genetic configurations. We noted that Cre recombination is often incomplete in our transgenic animals (Fig. 3b–e), similarly to a previous observation reported for a different Cre transgenic line^22^. Specifically, even though the F0 founder fish produced offspring expressing transgenesis marker Cold Heart, Cre-mediated reporter expression was often incomplete in the Cold Heart-positive offspring. The reasons for the inefficient recombination event could be due to 1) variegated Cre expression at either transcription or translation level, 2) inefficient recombinase activity of expressed Cre protein, 3) variegated expression of the *UAS:intersec* reporter line, or a combination of all three effects.

### Image registration and initial characterization of Gal4 patterns suggest new avenues into functional studies of neural circuits

**Figure 4:**
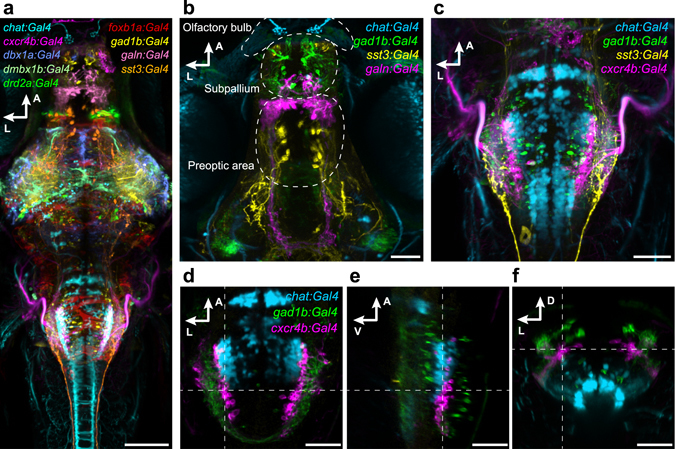
Registration of transgenic lines illustrates expression in distinct neuronal subpopulations. (**a**) Dorsal view on a reference brain showing the expression of selected, co-registered Gal4 lines. (**b**,**c**) Z-projection of confocal stacks showing the expression pattern of *chat:Gal4*, *gad1b:Gal4*, *sst3:Gal4 and galn:Gal4 or cxcr4b:Gal4*, respectively, in the deep fore-/midbrain (**b**), and in the hindbrain (**c**). (**d**–**f**) Single confocal slices of *chat:Gal4*, *gad1b:Gal4*, and *cxcr4b:Gal4* expression in the hindbrain, in dorsal (**d**), lateral (**e**) and cross-sectional view (**f**). Dashed lines indicate plane positions. Scale bar, 100 µm in (**a**) and 50 µm in (**b**–**f**).

In order to determine the three-dimensional labeling pattern of our newly generated Gal4 lines, we scanned optical sections at high resolution with a confocal microscope and performed image registrations across age-matched specimens^23^. By registering expression patterns to a standard reference brain, it is possible to compare the distribution of labeled cells with those present in other lines. This data format can be integrated in a comprehensive brain atlas^21, 24^. We found that the *HuC:lynTagRFP-T* marker is excellently suited as a bridging template for across-line image registrations. This membrane-targeted red fluorescent reporter is expressed in almost all neurons and strongly labels the cell membrane-enriched neuropil areas, whose outlines in the fish brain are highly stereotyped. After crossing Gal4 carriers of selected lines, driving the expression of green-fluorescent UAS-linked reporters (*UAS:Dendra*, *UAS:GFP*, or *UAS:GCaMP6s*), to carriers of the *HuC:lynTagRFP-T* transgene, triple-transgenic larvae were identified and whole-brain images of red and green channels were obtained. These images were aligned with each other using the *HuC:lynTagRFP-T* pattern as a template, and the green reporter channels were superimposed using different colors to visualize the spatial relationships of the respective patterns (Fig. 4a and Supplementary Video 12).

This analysis demonstrates the utility of our new lines for functional investigations into neural circuitry. Three immediate applications come to mind. First, telencephalic inhibitory connections have to our knowledge not been studied in teleosts. Our new *gad1b:Gal4* line will be particularly useful for functional studies of GABAergic cells in the subpallium, where the labeling seems particularly strong (Fig. 4b). Second, while the zebrafish olfactory bulb has been extensively investigated, recently culminating in the complete electron-microscopic reconstruction of its synaptic connectome^25^, considerably less is known about extrinsic modulation of olfactory processing. The new *chat:Gal4* line may give an entry point into this question, as it reveals the ventral pallial cholinergic innervation of the olfactory bulb (Fig. 4b). Lastly, neuropeptides have been shown to present highly specific markers of neurosecretory populations in preoptic and hypothalamic areas^17, 18^. Our new *sst3:Gal4* and *galn:Gal4* lines will allow recording and manipulation of these poorly understood modulatory systems.

### Image registration reveals new aspects of the layered architecture of the larval brain

Our initial characterization also revealed interesting architectonic principles that invite future investigations. We identified Gal4 lines with clustered, but mutually exclusive expression in the rhombencephalon (Fig. 4c). Specifically, *chat:Gal4*, *gad1b:Gal4* and *cxcr4b:Gal4* exhibited non-overlapping expression in rostral to caudal stripes in horizontal sections (Fig. 4d,e), and dorsomedial to ventrolateral stripes in transverse sections, respectively (Fig. 4f). This observation is consistent with the previously reported striped organization of neurotransmitter and transcription factor expression in the hindbrain^26^. Our analysis now shows that this developmental patterning not only holds true for glutamatergic, GABAergic, and glycinergic groups of neurons, as reported by Kinkhabwala *et al*.^26^, but also for cholinergic neurons. A multi-label image registration approach, such as the one used here, is uniquely suited to uncover principles of mesoscale spatial organization.

**Figure 5:**
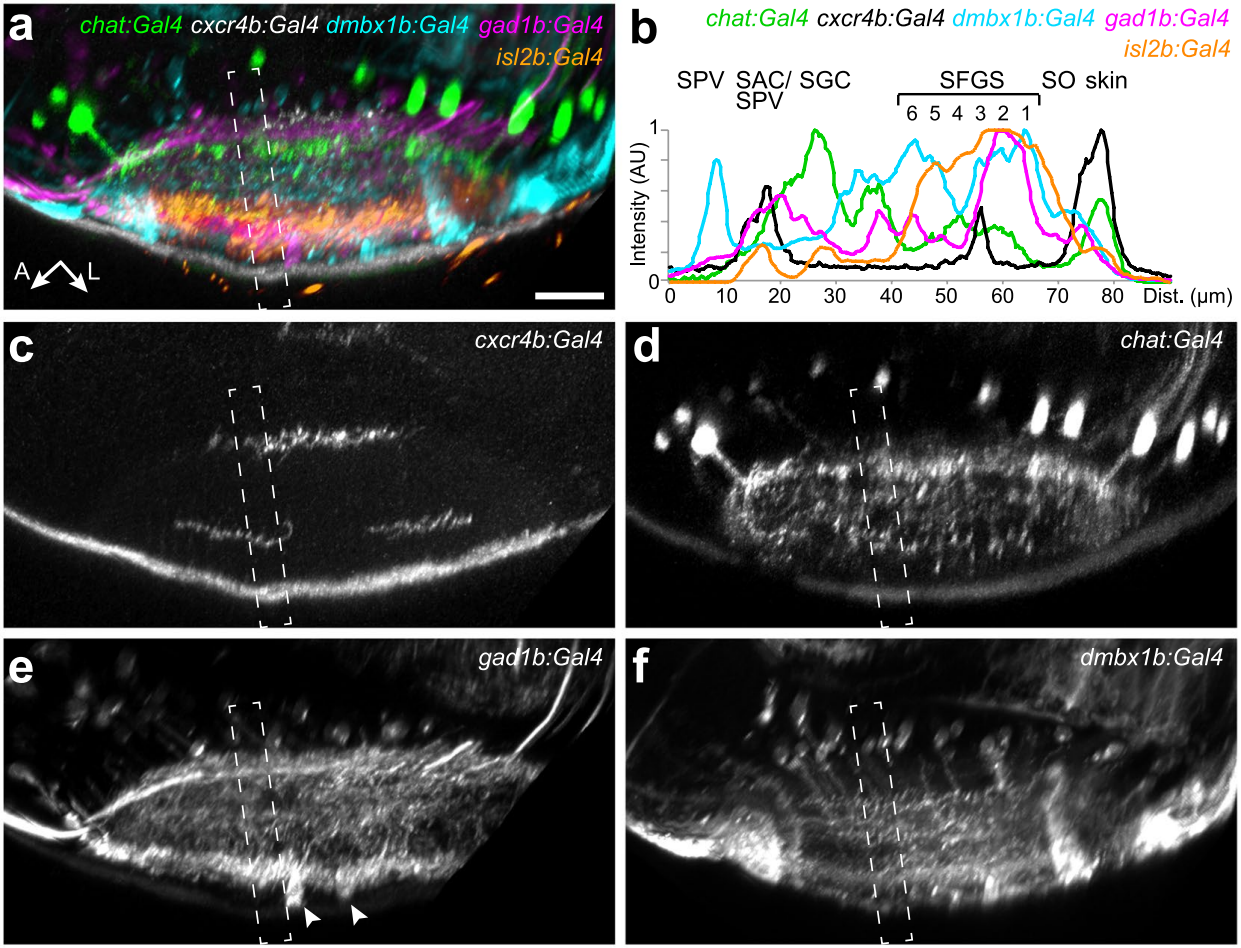
New transgenic lines label distinct sublaminae in the tectal neuropil. Lateral view of the tectal neuropil shows registered expression pattern of *chat:Gal4*, *cxcr4b:Gal4*, *dmbx1b:Gal4*, *gad1b:Gal4*, and *isl2b:Gal4*, as merged (**a**) or single channels (**c**–**f**). (**b**) Fluorescence intensity plots along the boxed regions in (**a**–**f**). Intensity peaks of *isl2b:Gal4* expression were used for layer determination. SIN cell bodies labeled by *gad1b:Gal4* are marked by arrowheads in (**e**). The peak for *dmbx1b:Gal4* in the SPV layer reflects labeled periventricular cell bodies.

The tectum is an intensively studied, multisensory processing area in the vertebrate midbrain^27^. Its densely packed neuropil is subdivided into layers, originally defined by the stratification pattern of incoming RGC axons^28–30^. RGC axons project to the following layers (from superficial to deep): Stratum opticum (SO); stratum fibrosum et griseum superficiale (SFGS, with its six sublayers, SFGS1 through SFGS6); the retinorecipient stratum griseum centrale (SGC); and the boundary between stratum album centrale and stratum periventriculare (SAC/SPV). From among our collection of BAC lines, several showed expression in the tectal neuropil (Fig. 5a). Co-registration of the patterns of our newly generated lines with that of an RGC-specific Gal4 line (*isl2b:Gal4*)^31^ allowed us to further explore the laminar architecture of the tectum beyond RGCs (Fig. 5a,b). A densitometric analysis revealed that *cxcr4b:Gal4* consistently but sparsely labeled SFGS3, SFGS4 and SAC/SPV (Fig. 5c). While the labeling of SFGS arises from *cxcr4b* + RGC axons, arborizations in the SAC/SPV layer originate from processes of deep midbrain neurons (data not shown). Neuropil labelings in *chat:Gal4*, *dmbx1b:Gal4* and *gad1b:Gal4* do not derive from RGC axons, but from neurites of specific classes of periventricular tectal neurons and incoming axons from various other brain areas (Fig. 5d–f). The *chat:Gal4* pattern showed highest fluorescence intensities in the deep stratum griseum centrale (SGC) (Fig. 5d). Fluorescence signals in *gad1b:Gal4*, on the other hand, are strongest in the superficial layers (Fig. 5e), including superficial interneuron (SIN) cell bodies, which are GABAergic^32^. Neurites labeled by *dmbx1b:Gal4* arborize in SFGS and SGC sublayers that are superficial to the retinorecipient SGC (Fig. 5f). As a rule, neuropil stratifications in these three lines are not restricted to single layers, but differences in labeling intensities rather reflect graded preferences for layer positioning. In summary, we isolated an array of transgenic lines, which allow targeting of different components of the layer-specific circuitry in the zebrafish tectum.

### Conclusions and outlook

Progress in the neurobiology of the zebrafish preparation has recently accelerated, driven by advances in imaging technology, behavioral analysis and computational methods^25, 33–39^. Optogenetics affords the ability to manipulate circuit components in the behaving animal^40–42^. We expect that our new set of BAC transgenic Gal4 and Cre lines will facilitate research that employs imaging or optogenetics in the zebrafish system and thus provide a valuable resource for the neuroscience community. In the future, breakthroughs are expected from the refinement of genetic tools that allow addressing specific subpopulations of neurons in the context of intact circuitry. Knock-in technology using CRISPR/Cas9 represents a promising approach to target endogenous loci with superior fidelity^43–46^ and may in the future be scaled up for the systematic generation of transgenic lines.

## Methods

### Animal care and transgenic lines

All animal procedures conformed to the institutional guidelines of the Max Planck Society and the local government (Regierung von Oberbayern). Experimental protocols were approved by Regierung von Oberbayern (55.2-1-54-2532-101-12 and 55.2-1-54-2532-31-2016). The previously described transgenic lines used in this study are as follows: *Tg*(*UAS:Dendra-kras*)*s1998t*; *Et*(*E1b:Gal4-VP16*)*s1101t*; *Tg*(*isl2b*.*3:Gal4-VP16*)*zc65*; *Tg*(*UAS:GCaMP6s*)*mpn101*; *Tg*(*5xUAS:EGFP*)*zf82*. These and all of the newly generated transgenic lines are available upon request.

### Plasmids

To generate pCR8GW-Gal4VP16-FRT-Kan-FRT-Bleeding Heart and pCR8GW-Cre-FRT-Kan-FRT-Cold Heart plasmids, the Bleeding Heart (cmlc2:mCherry, also known as myl7:mCherry) and Cold Heart (cmlc2:Cerulean, also known as myl7:Cerulean) cassettes (obtained from Michael Nonet, Washington University, USA) were inserted into pCR8GW-Gal4VP16-FRT-Kan-FRT and pCR8GW-Cre-FRT-Kan-FRT^16^, in the reverse orientation relative to Gal4VP16 or Cre coding sequence. To generate the Tol2 HuC:lynTagRFP-T plasmid, lynTagRFP-T (TagRFP-T tagged with lyn kinase membrane targeting sequence)^47^ was PCR amplified and cloned downstream of the HuC promoter. For the intersectional reporter, the loxP-tdTomato-CAAX-loxP-EGFP-CAAX cassette was codon optimized and synthesized by Genscript (Piscataway, NJ), and subsequently cloned into a pTol2-14xUAS vector to obtain UAS:loxP-tdTomato_CAAX-loxP-EGFP_CAAX.

### Selection of candidate genes and obtaining BAC clones

To identify corresponding BAC clones, we searched, using the Ensembl genome browser (www.ensembl.org/) or the UCSC genome browser (Zv6 assembly, https://genome.ucsc.edu/), for BAC clones that encompass both upstream and downstream of the first ATG site of the target gene. BAC clones were purchased from Source BioScience (www.lifesciences.sourcebioscience.com/) and BACPAC resources center, Children’s Hospital Oakland (https://bacpacresources.org/).

### BAC recombineering

BAC recombineering was performed as described previously^15, 16^. Briefly, in the first step, BAC clones were transformed with the *pRedET* plasmid (Gene Bridges), which enables the arabinose-inducible homologous recombination. In the second step, *Tol2* arms in opposing directions flanking an ampicillin resistance cassette were PCR amplified from *piTol2_amp* plasmid^16^ and inserted into the BAC backbone. In the third and final step, either *Gal4VP16* only, *Gal4VP16-BH* or *Cre-CH* cassettes were PCR amplified and inserted into the BAC, such that the start-ATG site of the gene of interest was replaced by that of *Gal4VP16* or *Cre*. For *th:Cre-CH* line, the GFP coding sequence of the *th:GFP* BAC^48^ was replaced with the *Cre-CH* cassette. After we confirmed successful insertions of the cassette by PCR, the final BAC DNA was purified using NucleoBond XTra BAC kit (Machery Nagel), and correct insertions were verified by sequencing.

### Transgenesis

*Tg*(*HuC:lynTagRFP-T*)*mpn404* ^49^ and *Tg*(*UAS:intersec*)*mpn128* were created using the standard *Tol2* transposon system. BAC DNAs were injected at 100 ng/µl together with zebrafish codon–optimized *Tol2* transposase mRNA (50–100 ng/µl), synthesized from pCS-zT2TP plasmid^16^. *Gal4VP16* and *Gal4VP16-BH* BAC DNAs were injected into *Tg*(*UAS:Dendra-kras*)*s1998t* transgenic embryos. *Cre-CH* BAC DNAs were injected into wild-type TL embryos. After injection, embryos expressing Dendra, Bleeding Heart, or Cold Heart were screened and raised to sexual maturity. Injected fish were either incrossed or outcrossed with wild-type or *Tg* (*UAS:Dendra-kras*)*s1998t* transgenic adult fish to identify transgenic carriers. The germline mosaicism rate was defined as a percentage of transgenic offspring (F1) out of all offspring born from F0 founder fish. Outcrossing of F1 transgenic lines (and of following generations) revealed segregation of Gal4 expression at Mendelian ratios, suggesting that they carry a single insertion. In cases where multiple founders were identified, the founders which showed the most complete expression patterns in their F1 offspring were selected and maintained. We noted that the transgene expression of different larvae derived from the same transgenic founders was variable in some of our BAC transgenic lines, as is known for the Gal4/UAS system in general^50, 51^. Nevertheless, among larvae that were pre-screened for the high-level expression of the transgene, the expression patterns were largely consistent across different larvae (Supplementary Figure S1).

### Immunohistochemistry

Immunostaining was performed according to ref. 24, with slight modifications. Fish were fixed in 4% paraformaldehyde (PFA) in PBS overnight. For the initial antigen retrieval, fish were heated to 63 °C for 15 minutes in 150 mM Tris-HCl. The time for staining with primary antibody was increased to at least 5 days and with secondary antibody to at least 2 days. Secondary antibodies were diluted in only PBT. After staining, samples were washed with PBT and postfixed for 30 minutes in 4% PFA, thereafter briefly washed in PBT and then soaked in 85% glycerol. C﻿﻿hAT antibody staini﻿ng was performed according to ref. 52﻿,﻿ with﻿ the protein﻿ase K treatment prolonged to 120 min for 6 dpf larvae. For a list of antibodies used, see Supplementary Table S1.

### Image acquisition

For live imaging, 6–7 dpf larvae were anesthetized in 0.016% tricaine and embedded in 2% low-melting-point agarose. Imaging was performed on a Zeiss LSM-780 or LSM-700 confocal microscope, using 20×/1.0 NA water-dipping objectives. For imaging fixed samples, larvae were embedded in 85% glycerol, and imaging was performed using a 25×/0.8 NA multi-immersion objective. Whole-brain images were acquired by tiling the brain into three individual image stacks and subsequent stitching, using ZEN software (black edition, v8.0; Zeiss). Images were corrected for fluorescence attenuation in the z-dimension using the brightness correction function in the ZEN software.

### Image registration

Registration was performed using the Computational Morphometry Toolkit (CMTK)^53^. Whole-brain images of living 6 dpf old zebrafish larvae were co-registered into one reference brain using expression of *HuC:lynTagRFP-T* as a template. For visualization of RGC innervation strata in the tectum, fish expressing *isl2b:Gal4 UAS:GCaMP6s* and *HuC:lynTagRFP-T* were co-registered into the reference brain.

## Electronic supplementary material

Supplementary Information

Supplementary Video 1

Supplementary Video 2

Supplementary Video 3

Supplementary Video 4

Supplementary Video 5

Supplementary Video 6

Supplementary Video 7

Supplementary Video 8

Supplementary Video 9

Supplementary Video 10

Supplementary Video 11

Supplementary Video 12
