# Supplementary material for: Genetic targeting and anatomical registration of neuronal populations in the zebrafish brain with a new set of BAC transgenic tools
Source: Sci Rep. 2017 Jul 12;7:5230. doi: 10.1038/s41598-017-04657-x (PMC5507991; doi:10.1038/s41598-017-04657-x)
Supplement: Supplementary file 1 — Supplementary Information [file 41598_2017_4657_MOESM1_ESM.pdf]

Supplementary Information

Genetic targeting and anatomical registration of neuronal populations in the zebrafish brain with a new set of BAC transgenic tools

Dominique Förster[1], Irene Arnold-Ammer[1], Eva Laurell[1], Alison Barker[1,2], António Miguel Fernandes[1], Karin Finger-Baier[1], Alessandro Filosa[1,2], Thomas Helmbrecht[1], Yvonne Kölsch[1], Enrico Kühn[1], Estuardo Robles[1,3], Krasimir Slanchev[1], Tod Thiele[1,4], Herwig Baier[1]* and Fumi Kubo[1]*

[1] Max Planck Institute of Neurobiology, Department Genes - Circuits - Behavior, Am Klopferspitz 18, D-82152 Martinsried, Germany

[2] Present address: Max-Delbrück Center for Molecular Medicine, Berlin, Germany

[3] Present address: Department of Biological Sciences, Purdue University, U.S.A.

[4] Present address: Department of Biological Sciences, University of Toronto Scarborough, Canada

*Correspondence should be addressed to H. B. (hbaier@neuro.mpg.de) and F. K. (fumikubo@neuro.mpg.de)

Supplementary Figure S1

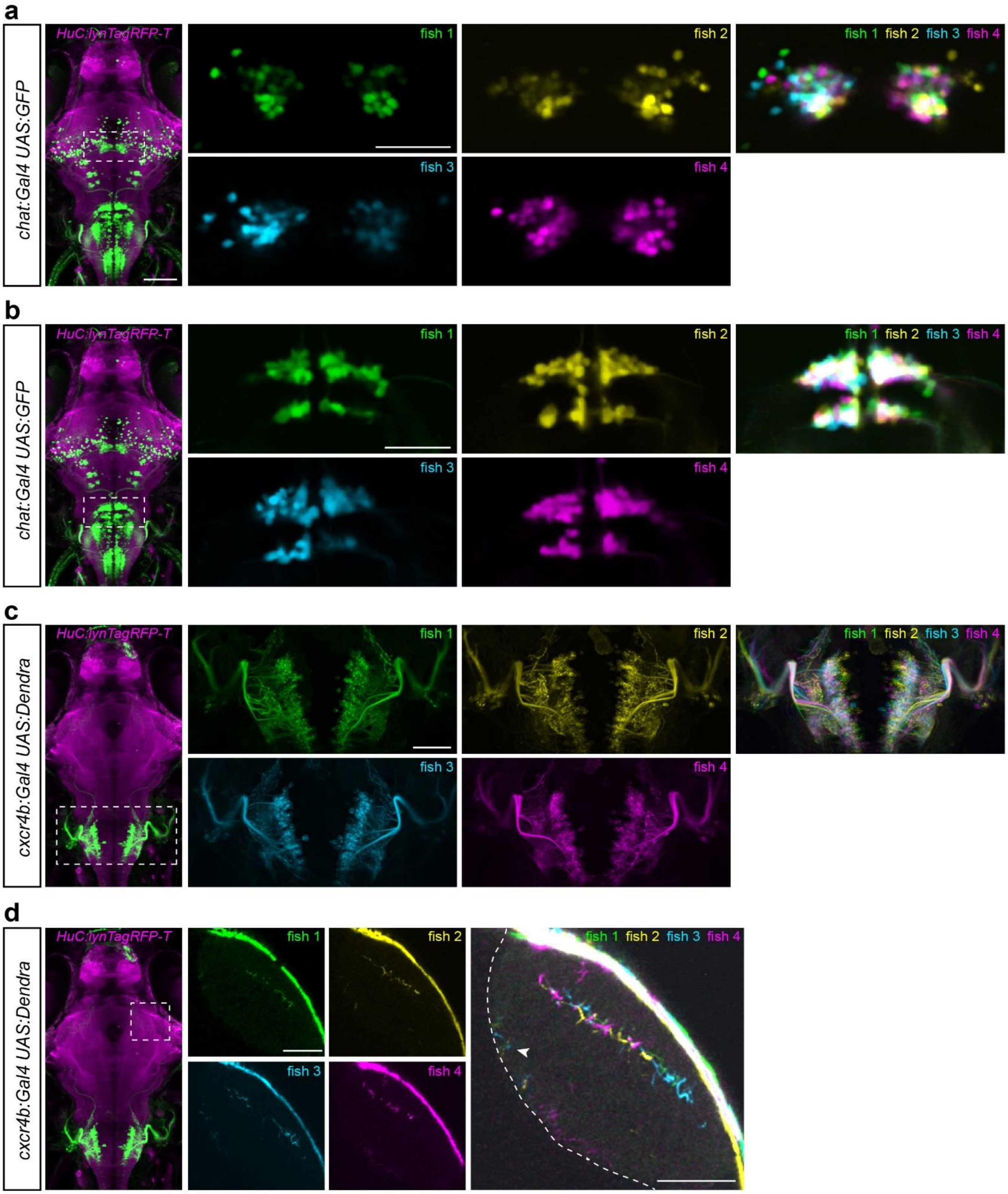

Supplementary Figure S1: Registration of different larvae of the same transgenic line illustrates variegated, but highly reproducible expression patterns. (a,b) Single confocal slices of *chat:Gal4* expression in extraocular motor nuclei (a) and hindbrain neurons (b). After sorting larvae for bright expression of the UAS reporter gene, four different larvae were imaged and co-registered using *HuC:lyn-TagRFPT* expression as a reference. **(c)** Z-projection of confocal stacks showing the expression pattern of *cxcr4b:Gal4* in the hindbrain. The overlay image reveals that consistent cell body position and axonal projections are largely consistent across four individual fish. **(d)** Single confocal slices of *cxcr4b:Gal4* expression in the tectum. An overexposed overlay is shown on the right. The labeled axons innervate the same layers (SFGS3 and SAC/SPV, arrow head) in the tectal neuropil in all four fish, although their precise location within the layers is variable between the four fish. Dashed line in the right panel indicates the border between the tectal neuropil and cell body layer. Scale bar, 100 µm (whole-brain images on the left) and 50 µm (close-ups).

Supplementary Table S1

**Primary antibodies**	**Source**	**Concentration**
Chick anti-GFP	Invitrogen A10262	1/250
Rabbit anti-Galanin	Merck Millipore AB5909	1/500
Rabbit anti-SST	ImmunoStar 20067	1/50
Rabbit anti-GABA	Sigma A2052	1/250
Mouse anti-HuC/D	Invitrogen A21271	1/250
Goat anti-ChAT	Merck Millipore AB144P	1/100

**Secondary antibodies**	**Source**	**Concentration**
Goat anti-chicken Alexa 488	Invitrogen	1/250
Goat anti-mouse Alexa 555	Invitrogen	1/250
Goat anti-rabbit Alexa 647	Invitrogen	1/250

Supplementary Table S1: List of antibodies used in this study.

Supplementary Video 1:

Confocal stack of *chat:Gal4* live expression pattern. See Figure 1 for details.

Supplementary Video 2:

Confocal stack of *cxcr4b:Gal4* live expression pattern. See Figure 1 for details.

Supplementary Video 3:

Confocal stack of *dbx1a:Gal4* live expression pattern. See Figure 1 for details.

Supplementary Video 4:

Confocal stack of *dmbx1b:Gal4* live expression pattern. See Figure 1 for details.

Supplementary Video 5:

Confocal stack of *drd2a:Gal4* live expression pattern. See Figure 1 for details.

Supplementary Video 6:

Confocal stack of *foxb1a:Gal4* live expression pattern. See Figure 1 for details.

Supplementary Video 7:

Confocal stack of *gad1b:Gal4* live expression pattern. See Figure 1 for details.

Supplementary Video 8:

Confocal stack of *galn:Gal4* live expression pattern. See Figure 1 for details.

Supplementary Video 9:

Confocal stack of *otx1b:Gal4* live expression pattern. See Figure 1 for details.

Supplementary Video 10:

Confocal stack of *sst3:Gal4* live expression pattern. See Figure 1 for details.

Supplementary Video 11:

3D volume representation of live expression in new Gal4 transgenic lines (green; Dendra-kras, GCaMP6s or EGFP). Brains have been registered via co-expression of *HuC:lynTagRFP-T* (magenta).

Supplementary Video 12:

3D volume representation of registered Gal4 expression patterns. Reporter channels of Supplementary Video 11 were superimposed with each other using different colors to visualize the spatial relationships of the respective patterns.
